# Hepatic radiofrequency ablation induces widespread cellular activation throughout the liver

**DOI:** 10.1186/s41747-026-00687-1

**Published:** 2026-04-02

**Authors:** Matthias Stechele, Justin Amadi, Lukas Salvermoser, Lorenzo Sperlich, Jonathan Monin, Daniel Khademi, Moritz Nikolaus Gröper, Muneeb Ahmed, Eithan Galun, Jens Ricke, Shraga Nahum Goldberg

**Affiliations:** 1 Department of Radiology, University Hospital, LMU Munich, Munich, Germany; 2 Goldyne Savad Institute of Gene Therapy and Division of Image-guided Therapy and Interventional Oncology, Department of Radiology, Hadassah Hebrew University Medical Center, Jerusalem, Israel; 3 Department of Medicine II University Hospital, LMU Munich, Munich, Germany; 4 Laboratory for Minimally Invasive Tumor Therapies, Department of Radiology, Beth Israel Deaconess Medical Center, Harvard Medical School, Boston, MA USA

**Keywords:** Liver, Mice, Radiofrequency ablation, Radiology (interventional), Single-cell gene expression analysis

## Abstract

**Objective:**

We investigated the extent of cellular, transcriptional, and translational activation throughout the liver following radiofrequency ablation (RFA).

**Materials and methods:**

RFA of the healthy liver was performed in two 8–10-week-old male C57/Bl6 mice, no/sham procedure in one. One and 7 days after, single-cell RNA sequencing (scRNAseq) was performed on distant, untreated liver to examine > 6,000 genes from normalized datasets of > 6,000 cells/sample, enabling identification of ten major cell populations. We defined cell-to-cell interactions by CellphoneDB and identified active pathways via STRING-db analysis with Markov clustering. Twelve distant liver lobe samples were homogenized on day 3 or day 6 after RFA/sham procedure for SomaLogic proteomic analysis (> 1,300 genes), subsequent STRING-db analysis, and assessment of cellular origin (PanglaoDB-2021).

**Results:**

CellphoneDB identified crosstalk among all ten populations with 4,658 and 4,218 receptor/ligand pairs, identified on day 1 and day 7 post-RFA, respectively. On day 1, 360 differentially expressed genes were identified; on day 7, 430. Activated genes distributed into 16 clusters, including 66 chemokines/cytokines, including Ccl2 and Ccl7; 57 immunomodulators, including Il6, Ctla4 and Pdcd1; and 54 growth factors, including Vegf, Hgf, Pdgf, and Fgf. Angiogenesis pathway genes were observed in endothelial cells and hepatocytes. Pdcd1 and Ctla4 were notably increased transiently in T cells. Proteomic analysis included 228/443 genes (51%) identified by scRNAseq; 73/228 proteins (32%) demonstrated 25% elevation over controls. Overall, 427 proteins were elevated, with 9/10 cell populations contributing to increased protein expression (odds ratio 4.9‒7.0).

**Conclusion:**

RFA diffusely activates cellular processes remotely from the ablation zone on both transcriptional and translational levels, altering tumorigenic and immunologic pathways simultaneously.

**Relevance statement:**

This study offers insights into liver tissue biology after RFA and provides a comprehensive picture of the molecular mechanisms put into motion by this procedure. A better understanding of these processes could provide a potential basis to develop specific biomarkers and effective adjuvant therapies following local tumor ablation.

**Key Points:**

- RFA activates a multiplicity of hepatic cellular processes remotely from the ablation zone on a transcriptional and translational level.
- Single-cell RNA sequencing provides insights into widespread cellular origins of activated pro-immunogenic, pro-tumorigenic, and other pathways detected post-ablation.
- Consideration of the nature of this response may help achieve the clinical goals of adjuvant therapies and predictive biomarkers.

**Graphical Abstract:**

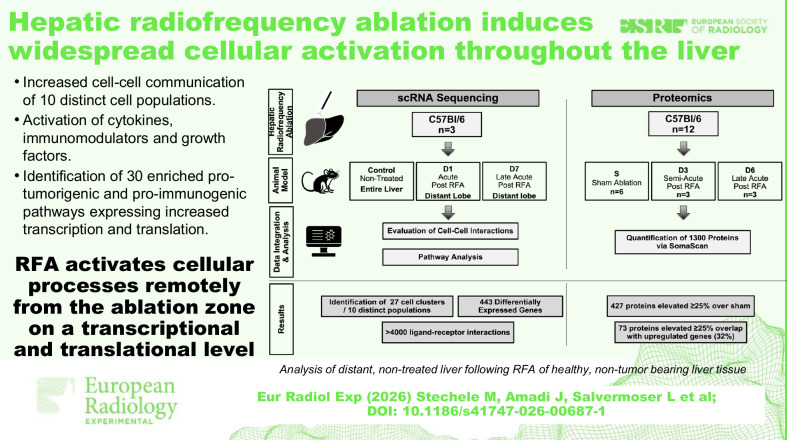

## Background

According to current guidelines of the European Society for Medical Oncology (ESMO), thermal tumor ablation is a first-line treatment option for hepatocellular carcinoma (HCC) [1, 2] and has recently been shown to be equally effective as surgical procedures for colorectal cancer in a randomized, controlled phase 3 trial [3].

To achieve successful hepatic tumor ablation, sufficient ablation margins extending beyond the tumor border are crucial. Therefore, a periablational zone of tissue coagulation at least 5 mm beyond the tumor border has generally been accepted as mandatory in every ablation procedure to minimize risk for local tumor progression [4, 5]. Thus, besides a central necrotic zone composed of coagulated cells, every ablation procedure inevitably creates a periablational zone composed of heat-stressed nontumorous tissue, containing native parenchymal hepatocytes, endothelial cells, and cholangiocytes [6]. Heat-stressed cell populations can initiate multiple pathways of reparative and reactive nature, locally recruiting fibroblasts and immune cell infiltration [7, 8]. Furthermore, ablation has been reported to activate many different downstream immune and inflammatory mechanisms—including the production of cytokines, activation of growth factors and inflammation-associated protein pathways, evolving dynamically over the first week post-ablation. These events may change the immune microenvironment, possibly rendering cancer cell killing by immune cells or enhance the killing effects of the immune cells. A substantial number of these have been linked to favorable pro-immunogenic and unfavorable pro-tumorigenic pathways [9–11].

Although some specific pathways have been elucidated, the overall, more complex picture of cell activation and gene expression initiated by ablation remains poorly characterized. Accordingly, we sought to perform Single-cell ribonucleic acid (RNA) sequencing (scRNAseq), a more comprehensive technical approach capable of elucidating gene alteration profiles in a large number of intrahepatic cell populations over time. Unlike common RNA bulk sequencing, scRNAseq potentially allows for the analysis of gene alterations on a cell-by-cell level, potentially providing insights into the cellular origin of various pathways [12]. Although bulk RNAseq has been used to elucidate some key active genes in the context of thermal ablation [13], to our knowledge, comprehensive transcriptional or translational analysis of hepatic cellular populations individually at the single-cell level using scRNAseq, especially from the perspective of increased cell communication, have yet to be performed. Identifying the exact cell types active by scRNAseq and the corresponding genetic alterations paves the way for targeted biomarker development and personalized adjunctive therapies following radiofrequency ablation (RFA).

Therefore, the purpose of this study was to comprehensively elucidate the transcriptional activation (*i.e*., transcriptional upregulation and increased predicted ligand–receptor crosstalk) of cell populations at early (24 h) and subacute (day 7) time points following RFA of healthy, nontumor bearing murine liver in the lobe far from the ablation site, followed by correlative proteomic, translational validation within this subacute timeframe. Specifically, we endeavored to answer the following questions: (1) What is the extent of activation of cellular activity in the normal host organ 1–7 days after RFA?; (2) What are the key transcriptional and translational profiles of these cells?; and (3) Are the molecular pathways identified related to pro-tumorigenic, pro-immunogenic, reparative, or other processes?

To accomplish this, we performed advanced bioinformatic analysis, including CellphoneDB analyses of scRNAseq transcriptional data from the distant lobe of normal mice post-RFA of normal liver and correlated this with proteomic studies to identify the most active cell populations, molecular pathways and cell-to-cell communications.

## Materials and methods

### Study design

In the first phase of this experimental study, scRNAseq was performed on distant, nontreated liver of male C57BL/6 wild-type mice, aged 8‒10 weeks. Two mice underwent standardized hepatic radiofrequency ablation (RFA) at 70 ± 2 °C (1 W/4 mA) for 5 min of healthy, nontumor-bearing liver and were sacrificed either on day 1 or day 7 after RFA to assess the acute and subacute transcriptional ablation effects on hepatic cell populations.

One control mouse received no treatment (*i.e*., served as control), allowing for the evaluation of the transcriptional profile of hepatic cell populations under physiologic laboratory conditions without intervention. To ensure high-quality scRNAseq data, the livers were perfused, and viable hepatocytes and nonparenchymal liver cells were isolated following surgical excision of the right liver lobe for subsequent scRNAseq analysis, which for day 1 and day 7 represented the distant, unablated lobe. In brief, the cells were isolated by perfusion and dissociation of the liver tissue and then purified and cleared of dead cells. Gene expression libraries were prepared, sequenced and the resulting data processed for scRNAseq analysis. Subsequently, quality assessment and cell population identification were performed. This was followed by evaluation of cell-cell interactions, using CellphoneDB and molecular pathway analysis, using the “Search Tool for the Retrieval of Interacting Genes/Proteins” database (STRING-db) [14] and databases in “Enrichr” [15].

In the second phase, validation of scRNA results using proteomics followed. Mice were assigned to four groups of three each for day 3 and day 6 for both RFA or sham procedure. Livers were harvested, distant liver lobes prepared as above, and frozen cellular suspensions, pooled for proteomic analysis (SomaScan®, SomaLogic Inc.) (1,300 protein analysis) as previously described [16]. Proteins elevated 25% over sham baseline were considered elevated based upon this threshold being shown as biologically relevant in the RF ablation system [9]. Additional analysis was performed at the 15% and 40% elevation, including determination of the top ten Gene Ontology Biological Process (GOPB) pathways for these thresholds overlapping with transcriptional activation via STRING-db. The two groups of sham had sufficiently similar protein expression to be pooled for the analyses.

Gene expression and protein data were compared to determine common pathways. 30 moderately sized (*i.e*., 49–545) pathways with high signal (> 5) and a false discovery rate (FDR) of < 10^-10^ on both the three largest gene clusters (*i.e*., combined cytokine/immune, growth factor, and collagen clusters) and corresponding protein clusters were analyzed. This included 18 Gene Ontology databases, 7 murine Reactome 20204, 3 mouse WikiPathways and 2 KEGG database pathways. For each pathway, the percentage of elevated genes and the associated signal, strength, and FDR were analyzed for RNA and protein expression separately and combined using STRING-db. Adjusted (Bonferroni-corrected) *p*-values, odds ratios (OR), and combined scores for these pathways were likewise calculated using the “Enrichr” website of databases for both the list of all 797 proteins identified by CellphoneDB and proteomics, as well as for the most restrictive scenario of the 73 genes common to both lists. Cellular origin of the proteins was assessed using the PanglaoDB 2021 in Enrichr [17]. Detailed information regarding the animal model, cell isolation and euthanasia, scRNAseq technique, and data processing can be found in the Supplementary tables and figures.

### Gene/protein analysis

The relationship of differentially expressed genes (DEGs) was evaluated by STRING-db pathway analysis [15] to define key active pathways and interactions. As default parameters, the following settings were applied: full STRING-db network, meaning of network “confidence,” minimum required interaction score: high confidence level: 0.700; Markov cluster algorithm‒MCL inflation parameter 2. The generated Markov clusters were included in the analysis for clusters containing at least 5 protein-coding DEGs and were referred to as “protein clusters.” One cluster specifically consisted of 123 DEGs and was therefore divided into two separate functional subclusters (*i.e*., cytokines and immunomodulators) using k-means clustering.

Heatmaps were generated for the largest families to express results graphically. To analyze individual gene expression per cell population and time point, first, feature counts for each cell were divided by the total counts for that cell. Normalized counts were scaled per gene across all cells using z-transform. Gene expression was measured by standard deviation. Alteration of gene expression between two time points was calculated as the difference between the scaled expressions and was displayed in colors ranging from red to blue. Dendrograms exhibiting genes were split for better visibility. Formally, k-means clustering was applied on the rows of the heatmap, where k was 3 or 4, thereby grouping DEGs with similar expression patterns across cell populations into “subclusters” A–D. Twelve representative, individual genes previously described in the literature as being elevated post-ablation [8, 11, 16, 18–20] and identified by our scRNAseq studies were assessed by stain plot to identify cellular expression in pictorial form.

## Results

### Initial determination of cell populations: scRNAseq analysis

A total of 27 clusters across the three samples were identified within the scRNAseq specimens, visualized using Uniform Manifold Approximation and Projection‒UMAP (Supplementary Fig. S1a). When combining cell numbers, cluster 0 contained the highest number of cells (*n* = 2,517), while cluster 26 exhibited the lowest (*n* = 50). All 27 clusters were assigned to 10 different cell populations representing the broad spectrum of cells found in the liver, including: parenchymal (hepatocytes), nonparenchymal liver cells (endothelial cells, Kupffer cells, hepatic stellate cells (HSCs), and cholangiocytes), as well as immune cells (B cells, T cells, natural killer (NK) cells, neutrophils, and macrophages) (Supplementary Fig. S1b). Overall, when combining the three time points, a total of 20,799 cells were detected (control, *n* = 7,365; day 1: *n* = 7,996; day 7: *n* = 5,438). Among the detected populations, endothelial cells were the highest represented cell population, *n* = 4,625 of 20,799 (22.2%), followed by macrophages, *n* = 3,557 (17.1%), and hepatocytes, *n* = 3,470 (16.7%). Full breakdown for each sample is provided as Supplementary Table S1.

### Increased cell-to-cell communication throughout the remote liver after RFA

Following cell population identification, we investigated the crosstalk between different cell types post-RFA at two time points, day 1 and day 7. Applying CellphoneDB, we noted substantial increases in cell-to-cell communication over control liver (Table 1). On day 1, the total number of ligand–receptor interactions, for all ten cell populations, was 4,658 (*p* = 2.70 E-61). The most active cell populations regarding cell-to-cell communication were HSCs (828), cholangiocytes (*n* = 761), and endothelial cells (*n* = 761). The most active intercellular communication between cell populations was HSCs communicating with cholangiocytes (*n* = 98), and cholangiocytes with endothelial cells (*n* = 79). Many cell populations displayed substantial auto-communication, *i.e*., the cell population communicating with other cells of like type, the most active being cholangiocytes (*n* = 84, Table 1a).

**Table 1:** Increased cell-to-cell communication in the distant, nontreated liver lobe on day 1 (D1) and on day 7 (D7) following hepatic radiofrequency ablation

a												
D1:	HSCs	Cholangiocytes	Endothelial cells	Macrophages	Kupffer cells	T cells	Neutrophils	NK cells	Hepatocytes	B cells	∑ Ligands	∑Ligands ∧ Receptors
HSCs	72	**98**	**77**	44	32	29	20	24	17	23	436	828
Cholangiocytes	**79**	**84**	**79**	44	33	25	17	19	26	10	416	821
Endothelial cells	**74**	60	66	42	32	21	25	29	17	16	382	761
Macrophages	50	42	49	53	37	23	23	23	14	14	328	615
Kupffer cells	37	35	36	32	24	13	15	13	12	12	229	456
T cells	21	16	15	17	16	6	5	8	10	8	122	264
Neutrophils	20	19	13	20	18	7	12	8	4	7	128	261
NK cells	18	15	15	13	14	7	4	7	7	4	104	249
Hepatocytes	10	27	19	12	13	6	8	10	2	8	115	225
B cells	11	9	10	10	8	5	4	4	1	7	69	178
∑ Receptors	392	405	379	287	227	142	133	145	110	109		

b												
D7:	HSCs	Endothelial cells	Cholangiocytes	Macrophages	Kupffer cells	Neutrophils	NK cells	Hepatocytes	T cells	B cells	∑ Ligands	∑ Ligands ∧ Receptors
HSCs	**102**	57	69	40	19	18	23	12	18	19	377	768
Endothelial cells	66	65	64	50	36	27	25	12	21	12	378	703
Cholangiocytes	**81**	46	57	34	18	15	16	20	14	17	318	623
Macrophages	39	54	31	49	42	25	23	10	21	13	307	579
Kupffer cells	30	31	22	26	22	13	18	10	15	10	197	405
Neutrophils	13	13	9	16	17	15	9	5	4	9	110	258
NK cells	17	13	13	12	12	9	4	7	2	4	93	239
Hepatocytes	19	23	20	18	22	10	12	6	6	12	148	238
T cells	19	19	13	16	12	9	9	7	8	6	118	233
B cells	5	4	7	11	8	7	7	1	6	7	63	172
∑ Receptors	391	325	305	272	208	148	146	90	115	109		

Bold values refer to the total number of individual cell-to-cell interactions ‘N’ exceeding the third quartile Q3: *N* > 73.75 and *N* > 76.75 which were also written in bold for Table 1a and 1b, respectively

Cell-to-cell communication between all detected cell populations was assessed by analyzing ligand–receptor interactions using CellphoneDB. Interactions with a *p*-value of 0 are reported. The matrix displays the number of increased cell-to-cell interactions on day 1 (D1) (**a**) and day 7 (D7) (**b**) in comparison to the control. Genes encoding proteins of the respective cell populations displayed horizontal function as ligands. Genes encoding proteins of the respective cell populations displayed vertical function as receptors. The total number of individual cell-to-cell interactions was divided into four different quartiles (Q)

Table 1a: *N* ≤ 25.25 (Q1); 25.25 < *N* ≤ 49.5 (Q2); 49.5 < *N* ≤ 73.75 (Q3); *N* > 73.75

Table 1b: *N* ≤ 26.25 (Q1); 26.25 < *N* ≤ 51.5 (Q2); 51.5 < *N* ≤ 76.75 (Q3); *N* > 76.75

*HSCs* Hepatic stellate cells, *NK* Natural killer cells

On day 7, the total ligand–receptor interactions, for all ten cell populations, were 4,218 (*p* = 5.43 × 10^-54^). The most active cell populations regarding cell-to-cell communication were HSCs (*n* = 768), endothelial cells (*n* = 703), and cholangiocytes (*n* = 623). The most active intercellular communication was between cholangiocytes with HSCs (*n* = 81) and endothelial cells with HSCs (*n* = 66). HSCs showed the most active auto-communication (*n* = 102, Table 1b).

Cell-to-cell communication was also altered in a time-dependent manner post-RFA. Hepatocytes were the only cell population with an overall increased number of interaction pairs from day 1 to day 7, increasing slightly from 225 to 238 (Δ = 13). All other cell populations showed fewer interaction pairs from day 1 to day 7, with the most notable decreases in cholangiocyte interactions, from 821 to 623 (Δ = 198), followed by HSCs from 828 to 678 (Δ = 60). Likewise, cholangiocyte auto-communication markedly decreased from *n* = 84 on day 1 to *n* = 57 interactions on day 7 (Δ = 27). By contrast, HSCs had substantially increased cell-to-cell interaction pairs from *n* = 72 on day 1 to *n* = 102 on day 7 (Δ = 30, Table 1).

### 443 genes demonstrate substantially increased expression after RFA clustered into 16 different protein groups

**Fig. 1:**
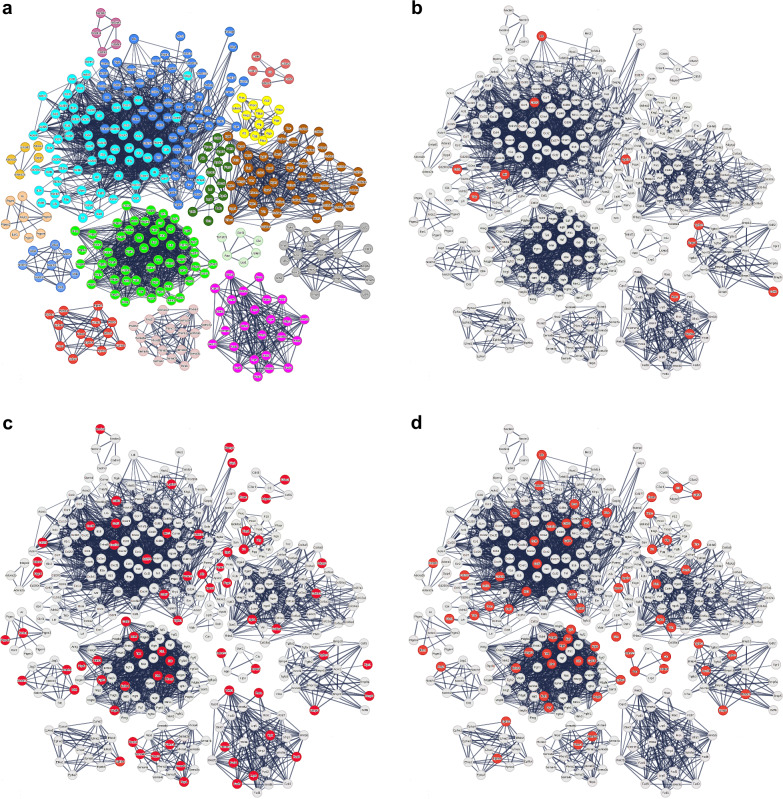
Mapping of transcriptionally differentially expressed genes (DEGs) in distant, nontreated liver tissue post radiofrequency ablation. STRING database (STRING-db) pathway analysis yielded 16 different clusters, each containing ≥ 5 DEGs per cluster. For better visibility, the intercluster associations are not depicted but can be seen at https://version-12-0.string-db.org/cgi/network?networkId=bxAsemOY9XkB. **a** In total, 365 DEGs are displayed as connected nodes and showed increased expression (log2 fold change ≥ 1 in at least 1 cell population; Markov cluster algorithm = 2) on day 1 (D1), day 7 (D7), or both, compared to control. DEGs of clusters with ≤ 4 DEGs (*n* = 49) and those not allocated to a cluster by STRING-db (*n* = 29) are not depicted. Each cluster has been assigned a different color. **b** DEGs with increased expression on day 1 (D1) exclusively (*n* = 13) are depicted as red nodes. **c** DEGs with increased expression on day 7 (D7) exclusively (*n* = 83) are depicted as red nodes. **d** 228 DEGs were assayed in a SomaLogic proteomics panel. Of these, 73 DEGs were translated with a ≥ 25% increase in fold change expression. These DEGs are depicted as red nodes (*n* = 66). An overview of the clusters can be found in Table 2

Although we had unfolded a pivotal global cellular interaction, to understand the meaning of this interaction, we needed to reveal the specific genes involved in these cell-to-cell communications and their kinetics. Accordingly, a gene of interest (GOI) list was constructed comprising 463 genes involved in at least one significantly increased cell-to-cell interaction at either day 1 or day 7 (Table 2). From these 463, 444 genes reached the predefined threshold of log2 fold change‒log2FC ≥ 1 either on day 1, day 7, or both in comparison to control for at least one cell population. One gene (mir9-3hg) was not identified by STRING-db and therefore excluded from further analysis, resulting in a total of 443 differentially expressed genes (DEGs). STRING-db analysis yielded a total of 15 different clusters of different functional protein groups containing at least 5 genes per cluster (https://version-12-0.string-db.org/cgi/network?networkId=b6bcn2IviYBg). Subdividing the largest cluster (*n* = 123) into cytokines (*n* = 66 genes), immunomodulators (*n* = 57) rendered 16 protein groups. The next largest groups were growth factors (*n* = 54) and genes associated with collagen production (*n* = 46, Fig. 1, Table 2).

**Table 2:** Overview of clusters of genes and proteins with increased expression

Cluster	STRING terminology RNA	RNA	Percentage out of 443 genes	STRING terminology protein	Protein	Percentage out of 398 proteins (427)overlapping with RNA clusters including percentage 228	73 overlap *N* genes			73 overlap *N* genes overlapping with protein clusters including percentage		
CYTOKINES	Viral protein interaction with cytokine and cytokine receptor	66	15	Cytokine-cytokine receptor interaction	43	11	11	45	24	13	43	30
IMMUNOMODULATORS	Mixed, incl. PI5P, PP2A and IER3 Regulate PI3K/AKT Signaling, and RET signaling	57	13	TNFR2 non-canonical NF-kB pathway	6	2	11	38	29	4	6	67
GROWTH FACTORS	Integrin cell surface interactions	54	12	Peptidyl-tyrosine phosphorylation	48	12	15	46	33	15	48	31
COLLAGEN	Non-canonical Wnt signaling pathway	46	10	ECM-receptor interaction	19	5	5	15	33	6	19	32
WNT	Positive regulation of pathway-restricted SMAD protein phosphorylation	25	6	Wnt, and Wnt-protein binding	5	1	0	2	0	0	5	0
TGFB/ACTIVIN/BMP	Semaphorin-plexin signaling pathway	20	5	TGF-beta signaling pathway	8	2	5	10	50	3	8	38
SEMAPHORIN	Plasma lipoprotein assembly, remodeling, and clearance	18	4	Axon guidance	5	1	2	6	33	4	5	80
LIPOPROTEIN	Complement and coagulation cascades	15	3	Cholesterol metabolism	6	2	3	7	43	1	5	20
FIBRINOLYSIS/COAGULATION	Ephrin receptor signaling pathway	13	3	Regulation of blood coagulation	17	4	3	10	30	3	17	18
EPHRIN	Notch signaling pathway	13	3	x	x	x	2	9	22	x	x	x
NOTCH SIGNALING	Intracellular steroid hormone receptor signaling pathway	8	2	x	x	x	1	7	14	x	x	x
PROSTAGLANDIN SYNTHESIS	Regulation of amyloid-beta clearance	8	2	x	x	x	1	2	50	x	x	x
AMYLOID/LIPOPROTEIN	Regulation of Complement cascade	6	1	High-density lipoprotein particle	15	4	4	5	80	5	15	33
COMPLEMENT ACTIVATION	Adenosine P1 receptors	6	1	Complement activation	19	5	2	2	100	2	19	11
NOREPINEPHRIN/ADRENALIN	Nectin/Necl trans heterodimerization	5	1	x	x	x	1	1	100	x	x	x
CELL ADHESION	X	5	1	x	x	x	0	2	0	x	x	x
CELLULAR SENESCENCE	x	x	x	Cellular senescence	32	8	x	x	x	2	32	6
MUSCLE PROTEIN	x	x	x	Muscle protein	9	2	x	x	x	0	9	0
PYRIMIDINE METABOLISM	x	x	x	Pyrimidine metabolism	7	2	x	x	x	1	7	14
CYTOSOLIC SMALL RIBOSOMAL SUBUNIT	x	x	x	Cytosolic small ribosomal subunit	6	2	x	x	x	0	6	0
GENES IN CLUSTERS WITH ≤ 4 GENES AS WELL AS UNCLUSTERED GENES		78	18	x	153	38	7	21	33	6	153	4

443 differentially expressed genes (DEGs) with ≥ double expression and 398 proteins with ≥ 25% increase in fold change expression were grouped into clusters according to the STRING database (STRING-db). Pathway analysis using STRING-db revealed 16 clusters for DEGs and 15 for proteins, with substantial overlap observed between the gene and protein clusters

Time-dependent dynamics: Of 443 DEGs, 347 (78%) were significantly increased in expression on day 1 and day 7. The cell populations with the greatest number of these 347 increased DEGs were hepatocytes (*n* = 180; 52%) and Kupffer cells (*n* = 81; 23%).

On day 1, 360 DEGs were detected, of which 13 DEGs were exclusively seen on day 1 (Fig. 1b). Although STRING-db analysis did not allocate these genes to more specific clusters, expression was most pronounced in endothelial cells and hepatocytes, where 8/13 genes were increased above a log2FC ≥ 1 threshold.

On day 7, 430 genes were detected. Of these, 83 DEGs were exclusively detected on day 7 (Fig. 1c). Alteration of these genes was most pronounced in hepatocytes, with 58/83 increased above the threshold of log2FC ≥ 1. Neutrophils (*n* = 31), NK cells (*n* = 30) and B cells (*n* = 29) also showed moderately increased expressed numbers of genes. STRING-db pathway analysis of these DEGs yielded 5 different functional protein groups: Sprouting Angiogenesis/Vascular Endothelial Growth Factor signaling, Wingless/Int‑1 family proteins Signaling, Lipoprotein Remodeling/Acute Phase reaction, Integrin-mediated interactions, and Tyrosine Kinase Signaling (Supplementary Fig. S2, https://version-12-0.string-db.org/cgi/network?networkId=bjafyr6QM75R). A breakdown of DEGs per cell population by gene cluster is presented as Supplementary Table S2.

### Analysis of transcriptional activation of three main protein groups by the cell population

**Fig. 2:**
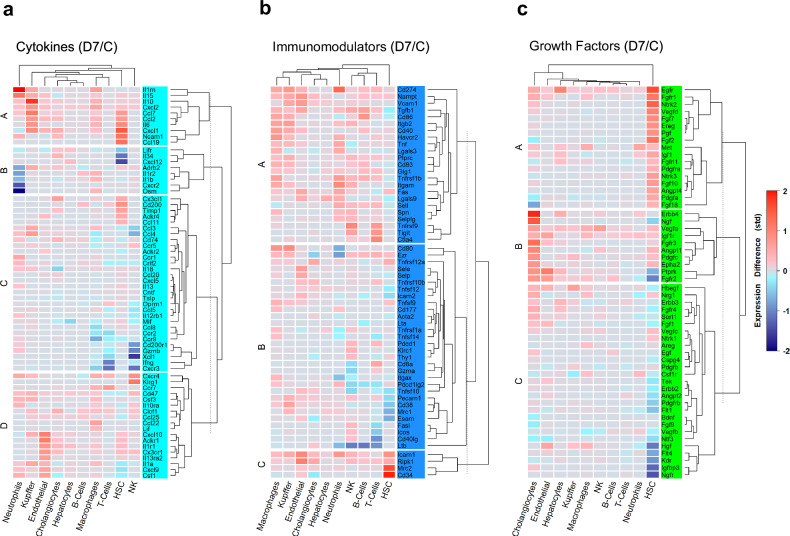
Heatmaps comparing day 7 (D7) post-ablation and controls (C) for differentially expressed genes (DEGs) represented in the three largest protein clusters across all cell populations. Dendrograms display cell populations, arranged from left to right in descending order, based on the combined expression differences of all families, which were clustered using k-means. **a** Expression differences of DEGs in the cytokine group. Overall, high expression differences for neutrophils as well as Kupffer cells were noted, with high expression for other cell populations in sub-families (such as hepatic stellate cells (HSCs) in A and endothelial cells in D). **b** For immunomodulators, DEGs in neutrophils and macrophages exhibited the highest expression differences, with virtually all cell populations showing high expression in subfamily A. **c** Expression difference of DEGs of the growth factor group is noteworthy, for DEGs in HSCs in subfamily A and cholangiocytes and endothelial cells in subfamily B showing the highest expression. Asterisks indicate genes that are displayed as stainplots in Fig. 3. NK, Natural killer cells; STD, Standard deviation

To further elucidate the potential relevance of the large number of DEGs and their cellular origin, Heatmaps of the three main protein clusters were constructed (day 7 results presented in Fig. 2, day 1 presented as Supplementary Fig. S3). For the cytokine cluster, overall, cell populations with the greatest expression increases were neutrophils, Kupffer cells, endothelial cells, and cholangiocytes. DEGs with increased expression differences in > 50% of cell populations included *Il10*, *Cxcl2*, *Cxcl10*, *Cd74*, *Clcf1*, and *Ccl25*. DEGs with increased expression in 50% of cell populations were *Il15*, *Ccl2*, *Ccl7*, *Cxcl1*, *Cxcr4*, and *Cx3cr1* (Fig. 2a).

In the immunomodulator cluster, neutrophils and macrophages had the greatest overall increases in expression, followed by Kupffer cells, NK cells, and T cells. Regarding individual genes, *Cd274*, *Nampt*, *Vcam1, Tgfb1* and *Icam1* had increased expression in most cell populations. Other immune checkpoint genes *Ctla4* and *Pdcd1* were increased in expression predominantly in T cells and NK cells (greater on day 7 than on day 1). When subdivided by hierarchical gene homology, substantial differences were noted in expression based upon cell population. For example, HSCs showed the least increased expression differences for subclusters A and B. Nevertheless, HSCs (and to a lesser extent, endothelial cells) showed increased expression in all four genes of the control group (Fig. 2b).

For the growth factor cluster, substantial variation in gene expression was observed among three subclusters (A–C, Fig. 2c). In group A, HSCs displayed the most expression increases, followed by hepatocytes; whereas cholangiocytes, followed by endothelial cells, had the greatest increases in groups B and C. Regarding individual genes, *Egfr* showed increased expression in all cell populations. *Met* was increasingly expressed in 6/10 cell populations. Other growth factors previously reported to be elevated after RFA (15) that showed increased expression in a substantial number of cell populations were *Fgfr1*, *Fgfr2*, and *Vegfa* (Fig. 2c).

**Fig. 3:**
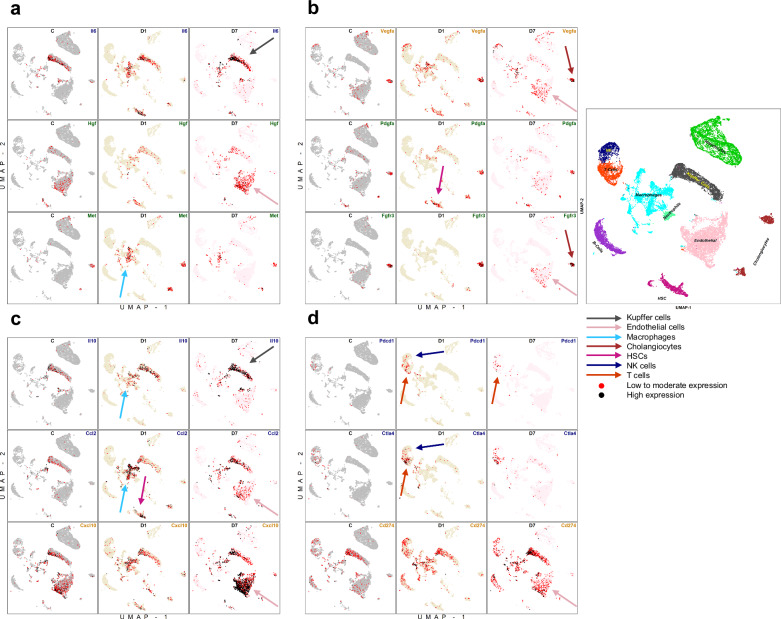
Stainplots of representative differentially expressed genes (DEGs) expressed across all cell populations. These include: (**a**) genes of the Il6/c-met/HGF pathway (5, 27), (**b**) additional growth factor genes reported elevated post-ablation (13, 15, 16), (**c**) known cytokines elevated post-ablation (25, 26), and (**d**) immunomodulators of known immunooncologic interest (23). Each figure consists of three different DEGs, arranged in rows. Additionally, each figure comprises three columns, which represent the different time points: Control (C) (left), day 1 (D1) (middle), and day 7 (D7) (right). Cells marked in red expressed the specified DEG at low to moderate levels (feature count *n* < 5), cells in black showed high gene expression (feature count *n* ≥ 5). To facilitate correlation of expression with cell population, each figure presented correlates to Supplementary Fig. S1. Colored arrows point to specific cell populations and time points with the greatest increased expression. An overview of all cell populations is provided. HSCs, Hepatic stellate cells; NK, Natural killer cells; UMAP, Uniform Manifold Approximation and Projection

To gain further insight into the relative expression of potentially relevant DEGs, we next constructed stainplots comparing the expression of 12 representative, highly expressed DEGs in control, day 1, and day 7 samples. For the four representative genes of the cytokine cluster, increased *Il10* expression was found in Kupffer cells and macrophages on both day 1 and day 7, as well as endothelial cells on day 7 (Fig. 3c). Likewise, *Ccl2* was increased most in Kupffer cells on day 1 and day 7. On day 1, *Ccl2* expression was highest in macrophages and HSCs. On day 7, *Ccl2* expression was likewise increased in endothelial cells and HSCs. *Cxcl10* was substantially expressed in Kupffer cells, endothelial cells, and cholangiocytes on day 7 (Fig. 3c). On day 1, HSCs, macrophages and Kupffer cells showed increased *Il6* expression. For macrophages, *Il6* gene expression was sustained to include day 7, with *Il6* expression increased even further for Kupffer cells (Fig. 3a).

As for immune checkpoints, similar findings were found for *Pdcd1* (CD274; PD-L1) and *Ctla4*, with increased expression noted in NK cells and T cells at both time points post-RFA, although decreased from day 1 to day 7. *Cd274* in general was expressed in all cell populations before and after RFA, with expression increased on day 1 in macrophages, NK cells, and neutrophils. On day 7, *Cd274* was increased in expression in every cell population except for cholangiocytes, hepatocytes, and HSCs (Fig. 3d).

For growth factor DEGs, substantial activity was observed in HSC and endothelial cells. Specifically, *Hgf* was increased in endothelial cells on day 7, whereas expression of *Met*, the receptor for the hepatocyte growth factor (HGF), was more pronounced on day 1 in macrophages and cholangiocytes (Fig. 3a). *Vegfa* had nearly ubiquitous increased gene expression across all cell populations on day 7. Increased expression of *Pdgfa* was found in HSCs on day 1. Endothelial cells and cholangiocytes exhibited the highest gene expression of *Fgfr3*, specifically on day 7 (Fig. 3b). Thus, heatmap and Stainplot data further demonstrate substantial robust transcriptional activity of multiple cytokines, immunomodulators, and growth factors across wide ranges of intrahepatic cell populations in the remote liver post-ablation.

### Multiple genes expressed by scRNAseq are translated to the protein level

To confirm the veracity and relevance of the observed increased transcription of the 16 protein groups, we performed SomaLogic proteomic analysis of tissue homogenates on day 3 and day 6. This assay included 228 of 443 genes identified by scRNAseq (51%). Of these, 73 proteins (32%) demonstrated 25% elevation over shams; 62 on day 3 and 15 on day 6 (Fig. 1d and Supplementary Fig. S4). Moreover, 31 proteins elevated at 40% increased expression had overlap with scRNAseq, and 103 proteins at 15% likewise overlapped with scRNAseq elevated genes (Supplementary Table S6). When analyzed by protein cluster, 11/45 (24%), 11/38 (29%), 15/46 (33%), and 1/15 (7%) proteins were detected for the cytokine, immunomodulator, growth factor, and collagen scRNAseq clusters, respectively (Table 2). Of the 83 genes elevated only on day 7, 13 genes (16%) showed increased translation.

**Fig. 4:**
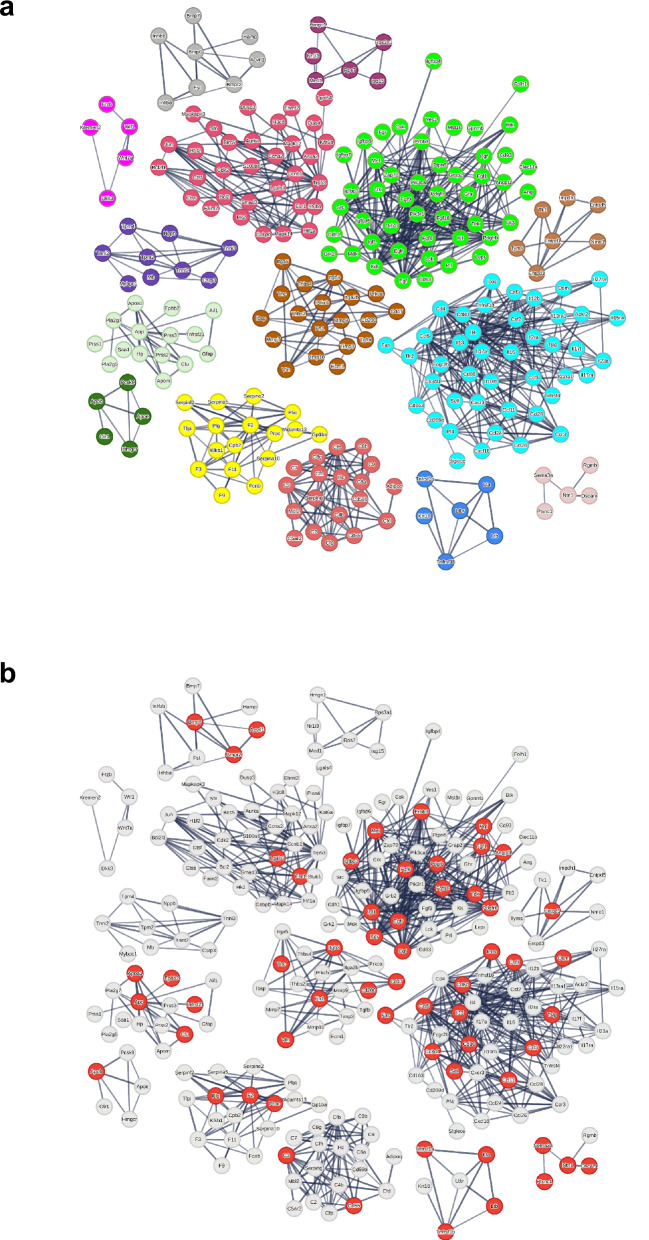
Mapping of proteins with ≥ 25% increased expression in distant, nontreated liver tissue post radiofrequency ablation. **a** In total, 245 proteins are displayed as nodes and showed increased expression (fold change ≥ 1.25; Markov cluster algorithm = 2) on either day 3 (D3), day 6 (D6) or both compared to sham ablation. Proteins that were not identified by the STRING database (STRING-db) are not displayed (*n* = 29). STRING-db pathway analysis yielded 15 different clusters, each containing ≥ 5 genes per cluster. Proteins in clusters with ≤ 4 genes per cluster (*n* = 69) as well as proteins that have not been allocated to a cluster according to STRING-db (*n* = 55) are excluded. Each cluster has been assigned a specific color in accordance with Fig. 3. **b** 73 proteins with a ≥ 25% increase in fold change, which were found to be among the differentially expressed genes identified at single-cell RNA sequencing, are depicted as red nodes (*n* = 59). For better visibility, the intercluster connections are not depicted but can be seen at https://version-12-0.string-db.org/cgi/network?networkId=bmu7gAt7lobs

When looking at the overall protein expression, a total of 427/1376 proteins (including the 73 above) demonstrated 25% elevation over shams; 330 (24%) on day 3 and 137 on day 6. STRING-db analysis identified 15 clusters of ≥ 5 proteins (https://version-12-0.string-db.org/cgi/network?networkId=bWKOi8ZlJ6Xj), of which substantial commonality (11/15) was noted in the scRNAseq data. The largest and second largest clusters corresponded to the growth factor and cytokine/immunomodulation clusters of scRNAseq, respectively (Fig. 4 and Table 2). Of note among newly identified clusters was “cellular senescence,” as well as several clusters of intracellular activity (filtered from the transcriptional data by CellphoneDB). Moreover, PanglaoDB identified all 10 cell populations as having significant contributions of increased proteins with ORs of 4.9‒7.0 for all cell populations except cholangiocytes (OR = 2.9) (Supplementary Table S3).

Finally, to further confirm the potential overlap of cytokinetic, immunomodulatory, growth factor and collagen molecular pathways between transcriptional and translational data, we performed molecular pathway analysis in 30 moderately sized representative pathways. These demonstrated strong correlations between transcriptional and translational data, as all selected pathways had marked enrichment (signal ≥ 13; FDR ≤ 1.7 × 10⁻⁴¹) on both the transcriptional and translational level, with 31 ± 9% (mean ± standard deviation) of possible pathway genes identified (Table 3 and Supplementary Table S4). Key immunogenic pathways with significant increase in both transcription and translation included “Regulation of IL[Interleukin]‑10 production” (GOBP: 0032653), “Chemokine-Mediated Signaling Pathway” (GOBP:0070098), and “Positive regulation of T-Cell Proliferation” (GOBP: 0042102; OR = 28.4, 27.8, 18.8, respectively). Multiple growth factor and tyrosine kinase pathways were similarly enriched, with markedly increased activation of angiogenesis and endothelial, epithelial, and fibroblast proliferation (OR > 12). Likewise, the top 10 GOPB pathways of the set of genes elevated on both transcription and translation (at the 40%, 25%, and 15% elevated protein level) confirmed these processes (Supplementary Fig. S5). Thus, multiple proteins associated with translated pathways are elevated after RFA in distant liver tissue.

**Table 3:** Pathway enrichment across transcriptomic, proteomic, and integrated datasets

			Total genes		RNA only (*N* = 443)		Protein only (*N* = 427)		String—combined RNA & protein (*N* = 797)					Enrichr—intersection RNA & protein (*N* = 73)		
Name of pathway	Classification	Pathway genes	*N*	%	*N*	%	*N*	%	*N*	% overlap	Strength	Signal	FDR	*p*-value*	OR	CS
Positive regulation of leukocyte migration	GO:0002687	176	66	37.5	52	29.5	25	14.2	11	16.7	2.09	20.77	6.99E-130	0.01591	21.6	115.7
Cytokine-mediated signaling	GO:0019221	371	90	24.3	61	16.4	38	10.2	9	10	1.77	15.48	1.88E-151	8.97E-08	14.3	286.2
Regulation of interleukin-10 production	GO:0032653	64	25	39.1	18	28.1	11	17.2	4	16	2.53	18.5	6.71E-58	0.0002717	27.5	292.3
Positive regulation of T-cell proliferation	GO:0042102	111	40	36	31	27.9	15	13.5	6	15	2.29	19.57	2.01E-85	0.0007711	18.9	175.2
Positive regulation of T-cell activation	GO:0050870	246	57	23.2	45	18.3	21	8.5	9	15.8	1.95	15.28	6.72E-105	0.003813	10.7	77.6
Chemokine-mediated signaling	GO:0070098	82	35	42.7	32	39	7	8.5	4	11.4	2.43	20.73	1.72E-78	0.0002852	26.8	283.2
Chemokine receptors bind chemokines	MMU-380108	49	30	61.2	27	55.1	7	14.3	4	13.3	2.65	23.1	1.61E-73	0.0004053	21.7	212.9
Signaling by interleukins	MMU-449147	262	46	17.6	19	7.3	31	11.8	4	8.7	1.92	13	3.42E-84	2.03E-08	10.6	221
Cytokine signaling in immune system	MMU-1280215	397	79	19.9	35	8.8	54	13.6	10	12.7	1.74	13.5	8.17E-132	4.25E-08	7.7	153.6
Cytokine-cytokine receptor interaction	mmu04060	280	129	46.1	94	33.6	55	19.6	20	15.5	1.89	24.73	4.36E-231	4.05E-18	27	1215.6
JAK-STAT signaling	mmu04630	165	44	26.7	21	12.7	31	18.8	8	18.2	2.12	16.9	3.49E-89	1.28E-06	15.8	250.1
Chemokine signaling	WP2292	174	42	24.1	27	15.5	19	10.9	4	9.5	2.1	15.98	1.68E-84	0.00918	6.7	36.9
Regulation of endothelial cell proliferation	GO:0001936	177	53	29.9	41	23.2	23	13	11	20.8	2.09	18.01	3.44E-104	4.77E-08	30.9	641.3
Transmembrane receptor protein tyrosine kinase signaling	GO:0007169	411	106	25.8	84	20.4	39	9.5	17	16	1.73	16.25	5.33E-174	2.25E-10	16.5	438.5
Growth factor activity	GO:0008083	152	59	38.8	47	30.9	24	15.8	12	20.3	2.16	21.12	7.42E-120	4.37E-14	48.8	1708.7
Fibroblast growth factor receptor signaling	GO:0008543	60	18	30	15	25	7	11.7	4	22.2	2.56	15.27	1.67E-41	0.002441	23	179.2
Positive regulation of MAPK cascade	GO:0043410	545	145	26.6	116	21.3	51	9.4	22	15.2	1.6	15.61	2.22E-221	1.89E-11	15.9	464.7
Positive regulation of angiogenesis	GO:0045766	180	56	31.1	47	26.1	23	12.8	14	25	2.08	18.45	9.26E-110	0.0007965	12	111.1
Positive regulation of epithelial cell proliferation	GO:0050679	239	67	28	54	22.6	31	13	18	26.9	1.96	17.26	3.75E-124	1.53E-12	36.8	1174.4
+ Reg of PI3-Kinase/Prot Kinase B signal transduction	GO:0051897	136	38	27.9	32	23.5	15	11	9	23.7	2.21	17.07	3.45E-78	3.92E-15	34.8	1336
Regulation of ERK1 and ERK2 cascade	GO:0070372	345	106	30.7	86	24.9	41	11.9	21	19.8	1.8	18.52	4.32E-181	6.52E-12	22.4	680.5
RAF/MAP kinase cascade	MMU-5673001	271	52	19.2	33	12.2	32	11.8	13	25	1.91	13.6	1.41E-94	6.15E-10	16.9	425
Signaling by receptor tyrosine kinases	MMU-9006934	410	102	24.9	70	17.1	49	12	17	16.7	1.73	15.82	1.49E-168	3.46E-11	12.3	346.4
Focal adhesion: PI3K-Akt-mTOR signaling**	WP2841	316	88	27.8	74	23.4	33	10.4	19	21.6	1.84	17.2	1.90E-155	2.03E-16	22.8	915.1
Integrin-mediated signaling	GO:0007229	92	25	27.2	21	22.8	8	8.7	4	16	2.38	15.97	1.65E-54	0.00001619	22.2	314.4
Extracellular matrix organization	GO:0030198	281	59	21	40	14.2	25	8.9	6	10.2	1.89	14.26	1.53E-105	0.1707***	3.1	6
Positive regulation of fibroblast proliferation	GO:0048146	78	26	33.3	22	28.2	13	16.7	9	34.6	2.45	17.56	2.14E-58	0.001058	34.1	302
Integrin cell surface interactions	MMU-216083	73	52	71.2	49	67.1	9	12.3	6	11.5	2.48	28.2	1.52E-119	4.74E-07	27	472.1
Extracellular matrix organization	MMU-1474244	247	90	36.4	69	27.9	29	11.7	8	8.9	1.95	20.72	2.55E-166	2.20E-07	12.1	220.7
Integrin-mediated cell adhesion	WP6	100	21	21	13	13	9	9	1	4.8	2.34	14.2	1.28E-46	0.3183***	2.8	3.3
Totals	Average	234.13	65.54	30.8	48.38	0.23	28.42	0.13	11.25	0.17	2.05	17.57			20.12	489.21
	STD	130.8	33.42	9.78	26.83	0.1	14.72	0.03	6.05	0.05	0.29	2.96			10.58	455
	Median												3.36E-105	4.51E-08		
	95% CI lower limit	178.88	51.43	26.67	37.05	0.19	22.2	0.11	8.7	0.15	1.93	16.32			15.87	297.08
	95% CI upper limit	289.37	79.65	34.93	59.7	0.28	34.63	0.14	13.8	0.19	2.17	18.82			24.37	681.34

30 moderately sized molecular pathways (49‒545 genes) from the cytokine, growth factor, and collagen clusters are presented. These show strong enrichment of the key pathways post-hepatic radiofrequency ablation with demonstrated homology between the transcriptional and translational data. STRING database (STRING-db) analysis is presented for all 797 enriched genes; whereas the last three columns present data for the most restrictive case of the 73 genes that were elevated at both single-cell RNA sequencing (scRNAseq) and proteomics. Additional analyses can be found in Supplementary Table S4

*N* Number of genes, *%* Percentage of total in group, *FDR* False discovery rate, *OR* Odds ratio, *CS* Combined score, *CI* Confidence interval, *STD* Standard deviation, *Strength* Enrichment strength score (from STRING-db), *Signal* Enrichment signal strength

* Adjusted *p*-value from Enrichr

** Enriched by STRING-db for both growth factor and collagen clusters

*** Significant for all 427 proteins (see Supplementary Table S4)

## Discussion

In this study, we found that RFA of nontumorous, healthy liver tissue, a mandatory requirement in any technically successful procedure, induces global activation on the cellular, transcriptional, and translational levels potentially throughout the entire untreated liver. This is manifested by increased cell-to-cell communication among all ten identified cell populations as observed through scRNAseq. Over 300 genes were activated on the transcriptional level 24 h after ablation. This increased to 430 by day 7, highlighting that gene alterations occur in a progressive and sustained fashion over the early post-ablation period. Moreover, this was associated with protein translation for a substantial number of diverse activated pathways.

The global activation we observed in growth factors on an RNA level in virtually every cell population is actively translated into proteins on day 3 and day 6 after RFA. A more comprehensive picture of increases and activation of multiple tyrosine kinases extending well beyond the previously reported vascular endothelial growth factor, HGF, platelet‑derived growth factor, transforming growth factor, and fibroblast growth factor (FGF) [19, 20] emerges. Thus, we hypothesize that a more general kinase inhibitor may be required for adequate adjunctive therapy to address the variety of pathways that are activated. Likewise, we note activation of multiple cytokine pathways, including IL-10 and positive activation and regulation of T cells. Thus, our findings suggest that hepatic RFA can induce simultaneous alterations in the genomic profile of markedly distinct protein groups, such as immunomodulation and growth, potentially inducing opposite clinical effects.

The 32% direct overlap between elevated RNA expression and corresponding 73 proteins is biologically meaningful for our complex system and can be considered strong supportive evidence for the presence of the identified molecular pathways, as prior researchers note this level of partial overlap as very good, due to post-transcriptional regulation, secretion or temporal lag [21]. Imperfect correlation can be attributed to a multiplicity of technical (*i.e*., pooled protein samples from multiple cells leading to a dilutional effect) as well as biologic reasons ranging from the fact that although many protein increases are transcriptionally driven, ~70% may involve, post-transcriptional regulation, delayed RNA-to-protein kinetics, non-coding regulation or translation control, and most likely a substantial element of protein secretion [22]. Indeed, key immunomodulatory and growth factor proteins, IL-10, IL-6, and FGF-2, that were seen on our single seq scanning, but not elevated in our protein analysis of tissue homogenates, have been observed in serum post-ablation [16]. Likewise, it is important to note that both the IL-6 and IL-10 receptor and multiple FGF family members were identified in our proteomic assays and that the IL-10 pathway and the “FGF leading to fibroblast proliferation pathway” were highly enriched. Thus, we likewise attribute our observation of a reduced number of proteins in the homogenate on day 6 (in the face of increasing transcription) to substantial secretion and active use by the tissue.

Given the severe extent of injury and stress induced by near coagulative doses of radiofrequency energy and heat to adjacent periablational tissues, it is not surprising that an intense reaction involving hundreds of genes results in activating a plethora of molecular pathways in most, if not all, cell populations. The liver apparently is recruiting the vast majority of native cell populations as well as multiple immune populations to combat the perceived insult. Yet, in the setting of malignancy (either micrometastases and pre-, if not malignant hepatocarcinogenesis), processes such as angiogenesis and growth factor production that lead to endothelial and fibroblast proliferation can have deleterious effects [20]. Likewise, the recruitment of macrophages and their conversion to the M2 state (as manifest by activation of mitogen‑activated protein kinase and extracellular signal‑regulated kinase pathways) can induce an unwanted pro-tumorigenic environment [23].

The largest number of genes transcribed were related to cytokines and immune processes. Immunomodulation and immune infiltration following local ablation have been increasingly researched in recent years [24, 25]. Our results, noting a significant increase in the expression of the receptor gene *Pdcd1* in T cells and NK cells, may further support a possible pro-tumorigenic, immunosuppressive receptor-ligand connection [26].

Our results regarding the increased expression of cytokines could have a direct impact on tumorigenesis, and subsequently, further studies: CXC motif chemokine ligand-10 plays an important immunomodulatory role across different tumor entities [27, 28]. Our results are also in concert with observations demonstrating the increased expression of *Cxcl10* in neutrophils after ablation and may indicate an attraction of specialized, anti-tumorigenic cells following the immediate stimulus after ablation. Additionally, high chemokine C-C motif ligand 2 expression is associated with poor survival in HCC and with increased infiltration of tumor-associated macrophages and fewer CD8+ tumor‑infiltrating lymphocytes [29].

IL-6 has been reported to be a potent initiator of the interlinked IL-6/HGF-c-Met/signal transducer and activator of transcription 3/vascular endothelial growth factor pathway, which was previously identified as a key driver of post-ablation distant tumorigenesis [8, 11, 18, 20, 30]. The current analysis also advances the understanding of the cellular origin of HGF post-ablation, as it was increasingly expressed in endothelial cells, macrophages, and Kupffer cells. Moreover, *MET* was not only increasingly expressed in hepatocytes, but also in macrophages and cholangiocytes one day after RFA, which indicates expression on an even broader basis originating from multiple cell populations. These findings enhance our understanding of previous literature, where a c-met inhibitor significantly attenuated HCC development in Multidrug Resistance Protein‒MDR-2 knockout mice treated with RFA [31]. Similar enhanced understanding of potentially relevant pathways emerges from our data, as we noted activation of multiple other pathways, such as WNT signaling and transforming growth factor β, that others have successfully inhibited to decrease tumor growth after ablation [32, 33].

Our analysis provides insights that are not feasible to obtain in clinical practice, where obtaining longitudinal tissue biopsies (especially of “normal” tissue) is ethically challenging. Thus, little and inconclusive evidence exists with regard to effects occurring distant to the treated site, with existing evidence mainly focusing on tumor-tumor interactions following ablation [34, 35], highlighting the complexity of the tumor type, organ microenvironment and timing at which the greatest immune activity occurs after local thermal treatment [36].

Our study has several limitations. Given the extensive set of cellular, transcriptional, translational and molecular processes observed, despite our initial intention of achieving a comprehensive understanding, our study is by no means exhaustive in identifying all active molecular pathways involved in the post-ablation process. We readily acknowledge that our approach precluded identifying smaller even more precise pathways with higher signal or odds ratios. We furthermore acknowledge the lack of biological replicates in the scRNAseq analysis, limiting assessment of inter-individual variability, even as we stress the more generalized acceptability of single samples for scRNAseq given the large number of reads. As a major limitation of this study, by studying the distant liver to assess effects there, we were not able to identify key pathways and cells in the periablational rim. Likewise, this initial study did not study the effects of ablation directly on micrometastatic or HCC tumors—future studies we intend to complete. Nevertheless, the wealth of data presented herein represents an important step toward understanding host responses *versus* tumor-related alterations in the post-RFA state. Further study is thus likely warranted, including extending to other forms of ablation (both thermal and other) and for other tumor microenvironments such as the kidney and lung. Although we predict similarities in response regarding observing processes of tissue repair and inflammation, accompanied by pro-immunogenic and pro-tumorigenic pathways, the precise nature will likely vary based upon tissue and tumor type and potentially further vary based upon the type of ablation performed.

Additional limitations of our work include the fact that the day 3 and 6 time points for protein analysis were not identical to the day 1 and day 7 post-ablation scRNAseq, and that our proteomics only assessed for one-tenth of the scRNAseq genes. Nevertheless, this can also be viewed as a confirmatory strength, as a substantial overlap and convergence in genes on the RNA and protein level and their pathways independently verified from three databases, analyzed on two website platforms, speaks to the strength of our overall observations. Indeed, we stress that analysis of proteins at two days after initial scRNAseq analysis secures insights into translational efficiency and thus provides a more physiologically relevant readout of the functional state of the liver and reduces potential over-interpretation of short-term, biologically irrelevant alterations.

We further acknowledge that the thresholds selected for defining elevation of genes, proteins and clustering can influence the outcome by altering the number of genes defined as elevated, as well as influence cluster/pathway assignment. Thus, although we were able to identify global transcriptional and translational activity throughout all cell populations in multiple highly enriched pathways, further future refinements and analyses will undoubtedly uncover additional relevant genes, proteins, and pathways involved in the post-ablation process.

In conclusion, our study demonstrates that RFA activates a multiplicity of hepatic cellular processes remotely from the ablation zone on a transcriptional and translational level. Among these hundreds of genes with increased expression, we moreover confirm the activity of many previously reported pro-immunogenic and pro-tumorigenic molecules and pathways, importantly noting that these can be activated simultaneously and are highlighted by the fact that there is a substantial overlap between transcription and translation, which is sustained for scores of genes over the day 7 post-RFA period. Thus, consideration of the complexity of robust responses will be necessary to achieve the clinical goals of developing effective adjuvant therapies and predictive biomarkers. Ideally, further research will identify a hierarchy of interaction among these, elucidating which molecules and pathways should be prioritized for targeting to enable optimal outcomes after interventional oncologic treatment.

## Supplementary information

Additional file 1:**Table S1** Full breakdown of cell counts and percentages of cell populations for each sample, control (C), day-1 (D1) and day-7 (D7). **Table S2** Differentially Expressed Genes (DEGs) with increased expression by cell population on day-1 (D1) and day-7 (D7). **Table S3** Enrichment analysis of cell populations. **Table S4** Enrichment of pro-tumorigenic and proimmunogenic pathways across RNA, protein, and integrated RNA and protein datasets. **Table S5** Identification of cell populations using gene markers. **Table S6** Threshold robustness of proteomic analysis with sensitivity results and Wilson 95% CIs for proportions elevated at 15%, 25%, and 40%. **Fig. S1.** Identification of cellular populations within the single-cell RNA sequencing samples as visualized by Uniform Manifold Approximation and Projection (UMAP). (**a**) 27 cellular clusters were identified across the three samples (0-26) arranged in descending order according to the number of cells. (**b**) These clusters were grouped into 10 cell populations based upon established gene markers (see Table S5). *HSC* Hepatic stellate cell, *NK* Natural killer cells. **Fig. S2.** STRING database pathway analysis of differentially expressed genes for new genes identified with increased expression on day-7 (D7) was performed on genes with increased expression (log2 fold change ≥ 1 in at least 1 cell population on day-7 (D7) exclusively (*n* = 83). Clustering yielded 5 clusters with a total of 37 genes in different functional protein groups, each containing ≥ 5 genes per cluster. The remaining 46 genes did not yield additional separate clusters of ≥ 5 genes or were not clustered at all and are therefore excluded from depiction. **Fig. S3.** Heatmaps comparing day-1 (D1) post-ablation and controls (C) for differentially expressed genes (DEGs) represented in the three largest protein clusters across all cell populations. Dendrograms display cell populations, arranged from left to right in descending order, based on the combined expression differences of all families, which were clustered using k-means. (**a**) Expression differences of DEGs in the cytokine group. (**b**) Expression differences of DEGs for the immunomodulators. (**c**) Expression differences of DEGs of the growth factor group. Noteworthy, DEGs in hepatic stellate cells (HSCs) in subfamily C showed the highest expression differences regarding growth factors. *NK* Natural killer cells, *STD* Standard deviation. **Fig. S4.** Color-coded STRING database mapping of 73 genes elevated on both a transcriptional (single-cell RNA sequencing) and translational (proteomics) level. Markov clustering demonstrates a large number of growth factors (light green) highly associated with pro-tumorigenic angiogenesis, fibroblast, and endothelial proliferation pathways. Multiple proimmune associated clusters are simultaneously identified including cytokines (light blue), immunomodulators (orange), and tumor necrosis factor/interferon regulation (yellow). **Fig. S5.** STRING database mapping of the top ten gene ontology biological process (GOBP) pathways of overlapping proteomic and single-cell RNA sequencing results using 15% (**a**), 25% (**b**), and 40% (**c**) threshold of increased protein expression. The GOBP pathways for these thresholds show remarkable similarity to other pathways previously analyzed. using the threshold of log2 fold change ≥ 1 in at least 1 cell population.

## Data Availability

Raw data were generated at Goldyne Savad Institute of Gene Therapy, Hadassah Hebrew University Medical Center, Jerusalem, Israel. Derived data supporting the findings of this study and subsequent analyses are available from the corresponding author (MSt) on reasonable request. Part of these data were derived from the following resources available in the public domain: STRING: functional protein association networks. © STRING Consortium 2025. https://string-db.org. Accessed 24 Aug 2023. Enrichr—Ma’ayan Laboratory, Computational Systems Biology, New York. https://maayanlab.cloud/Enrichr/. Accessed 29 Apr 2024. All data derived from STRING-db can be accessed via the cited permalinks.

## Abbreviations

DEG: Differentially expressed gene
FDR: False discovery rate
FGF: Fibroblast growth factor
GOBP: Gene Ontology Biological Process
HCC: Hepatocellular carcinoma
HGF: Hepatocyte growth factor
HSCs: Hepatic stellate cells
IL: Interleukin
NK: Natural killer
OR: Odds ratio
RFA: Radiofrequency ablation
RNA: Ribonucleic acid
scRNAseq: Single-cell RNA sequencing
STRING-db: Search Tool for the Retrieval of Interacting Genes/Proteins database
