# Supplementary material for: Hepatic radiofrequency ablation induces widespread cellular activation throughout the liver
Source: Eur Radiol Exp. 2026 Apr 2;10:38. doi: 10.1186/s41747-026-00687-1 (PMC13047045; doi:10.1186/s41747-026-00687-1)
Supplement: Supplementary file 1 — Additional file 1:Table S1 Full breakdown of cell counts and percentages of cell populations for each sample, control (C), day-1 (D1) and day-7 (D7). Table S2 Differentially Expressed Genes (DEGs) with increased expression by cell population on day-1 (D1) and day-7 (D7). Table S3 Enrichment analysis of cell populations. Table S4 Enrichment of pro-tumorigenic and proimmunogenic pathways across RNA, protein, and integrated RNA and protein datasets. Table S5 Identification of cell populations using gene markers. Table S6 Threshold robustness of proteomic analysis with sensitivity results and Wilson 95% CIs for proportions elevated at 15%, 25%, and 40%. Fig. S1. Identification of cellular populations within the single-cell RNA sequencing samples as visualized by Uniform Manifold Approximation and Projection (UMAP). (a) 27 cellular clusters were identified across the three samples (0-26) arranged in descending order according to the number of cells. (b) These clusters were grouped into 10 cell populations based upon established gene markers (see Table S5). HSC Hepatic stellate cell, NK Natural killer cells. Fig. S2. STRING database pathway analysis of differentially expressed genes for new genes identified with increased expression on day-7 (D7) was performed on genes with increased expression (log2 fold change ≥ 1 in at least 1 cell population on day-7 (D7) exclusively (n = 83). Clustering yielded 5 clusters with a total of 37 genes in different functional protein groups, each containing ≥ 5 genes per cluster. The remaining 46 genes did not yield additional separate clusters of ≥ 5 genes or were not clustered at all and are therefore excluded from depiction. Fig. S3. Heatmaps comparing day-1 (D1) post-ablation and controls (C) for differentially expressed genes (DEGs) represented in the three largest protein clusters across all cell populations. Dendrograms display cell populations, arranged from left to right in descending order, based on the combined expression differ [file 41747_2026_687_MOESM1_ESM.pdf]

Hepatic radiofrequency ablation induces widespread cellular activation throughout the liver

ELECTRONIC SUPPLEMENTARY MATERIAL

Table S1.

Day (count)	Hepatocytes	Kupffer cells	NK cells	T cells	Neutrophils	Macrophages	B cells	HSCs	Cholangiocytes	Endothelial cells	∑
**C**	2652	1029	156	284	30	452	305	89	223	2145	7365
**D1**	251	803	685	916	274	2564	972	933	423	175	7996
**D7**	567	851	126	258	53	541	543	96	98	2305	5438
∑	3470	2683	967	1458	357	3557	1820	1118	744	4625	20799
**Day (%)**	**Hepatocytes**	**Kupffer cells**	**NK cells**	**T cells**	**Neutrophils**	**Macrophages**	**B cells**	**HSCs**	**Cholangiocytes**	**Endothelial cells**	∑
**C**	36	14	2.1	3.9	0.4	6.1	4.1	1.2	3	29.1	100
**D1**	3.1	10	8.6	11.5	3.4	32.1	12.2	11.7	5.3	2.2	100
**D7**	10.4	15.6	2.3	4.7	1	9.9	10	1.8	1.8	42.4	100
**Mean**	16.7	12.9	4.6	7	1.7	17.1	8.8	5.4	3.6	22.2	100

Full breakdown of cell counts and percentages of cell populations for each sample, control (C), day-1 (D1) and day-7 (D7). *HSCs* Hepatic stellate cells, *NK* Natural killer cells.

Table S2 Differentially Expressed Genes (DEGs) with increased expression by cell population on day-1 (D1) and day-7 (D7).

a

CELLTYPE	N GENES INCREASED	CYTOKINES	IMMUNOMODULATORS	GROWTH FACTORS	COLLAGEN	WNT	TGFB/ACTIVIN/BMP	SEMAPHORIN	LIPOPROTEIN	FIBRYNOLYSIS/COAGLUTION	EPHRIN	NOTCH SIGNALING	PROSTAGLANDIN SYNTHESIS	AMYLOID/LIPOPROTEIN	COMPLEMENT ACTIVATION	NOREPINEPHRIN/ADRENALIN	CELL ADHESION	GENES ABSENT IN 16 MAJOR CLUSTERS
**Endothelial Cells**	225	45	35	23	28	13	8	5	6	5	6	4	4	3	2	2	2	34
**Hepatocytes**	218	37	25	29	23	10	11	8	8	7	5	3	3	3	3	2	0	42
**Kupffer Cells**	131	27	17	18	18	2	6	4	2	2	4	2	2	1	1	1	1	23
**B cells**	103	19	17	12	13	3	5	4	3	2	2	2	3	1	1	0	2	14
**HSC**	96	23	8	13	19	7	3	1	2	2	2	0	2	1	1	2	0	10
**Cholangiocytes**	94	21	14	10	15	1	2	1	1	3	3	0	3	0	2	2	0	16
**Macrophages**	79	16	7	10	16	4	1	2	0	1	3	1	3	2	1	2	0	10
**NK Cells**	74	17	9	8	15	2	0	4	1	3	1	1	1	0	0	1	0	11
**T cells**	72	14	11	7	14	3	3	1	4	0	2	2	0	0	2	0	0	9
**Neutrophils**	56	8	9	4	7	2	3	0	3	3	0	1	3	1	1	0	1	10

b

CELLTYPE	N GENES INCREASED	CYTOKINES	IMMUNOMODULATORS	GROWTH FACTORS	COLLAGEN	WNT	TGFB/ ACTIVIN/ BMP	SEMAPHORIN	LIPOPROTEIN	FIBRYNOLYSIS/ COAGLUTION	EPHRIN	NOTCH SIGNALING	PROSTAGLANDIN SYNTHESIS	AMYLOID / LIPOPROTEIN	COMPLEMENT ACTIVATION	NOREPINEPHRIN/ADRENALIN	CELL ADHESION	GENES ABSENT IN 16 MAJOR CLUSTERS
**Hepatocytes**	311	46	36	43	34	15	15	16	6	8	10	5	3	5	6	4	2	58
**Kupffer Cells**	158	32	18	18	16	4	6	5	6	5	5	2	3	2	3	3	2	28
**Cholangiocytes**	142	30	20	17	14	3	3	3	4	4	5	2	4	0	1	1	1	31
**B cells**	140	18	20	19	18	6	7	13	6	4	4	4	1	1	1	0	2	16
**Macrophages**	127	18	12	18	18	5	3	4	5	7	7	1	1	2	1	2	1	23
**NK Cells**	123	12	11	13	15	7	2	9	4	6	4	3	3	2	2	2	4	24
**HSC**	119	23	12	17	21	6	6	3	2	3	3	2	2	1	1	1	1	15
**Neutrophils**	111	18	16	7	11	1	2	5	7	9	3	2	3	2	3	1	1	20
**Endothelial Cells**	102	18	24	11	13	3	2	1	3	5	2	1	2	0	1	1	2	13
**T cells**	84	11	9	12	8	4	2	2	4	6	3	1	2	1	2	0	1	16

DEGs on day-1 (D1) (**a**) and day-7 (D7) (**b**) were identified in each cell population and grouped according to protein clusters identified by STRING database (STRING-db). The last column indicates the number of genes in each cell population which were not clustered by STRING-db. *HSCs* Hepatic stellate cells. *NK* Natural killer cells.

Table S3 Enrichment analysis of cell populations.

Cell type	Overlap	% overlap	Adjusted P-value	Odds Ratio	Combined Score
HSCs	17/132	12.9	3.02E-08	7.0	137.0
Hepatocytes	27/221	12.2	2.36E-11	6.7	195.1
Neutrophils	18/151	11.9	3.16E-08	6.4	124.4
Macrophages	24/204	11.8	5.29E-10	6.4	160.7
Kupffer cells	16/137	11.7	2.08E-07	6.3	106.7
NK cells	18/157	11.5	5.65E-08	6.2	115.1
B cells	20/182	11.0	2.24E-08	5.9	117.1
T cells	18/165	10.9	9.87E-08	5.8	104.2
Endothelial cells	23/246	9.3	3.02E-08	4.9	96.2
Cholangiocytes	8/134	6.0	0.0153	2.9	14.2

Potential cellular origin of 427 proteins increased post radiofrequency ablation (RFA) in distant liver homogenate. All 10 cell populations showed increased Odds Ratios and statistically significant present of overexpressed genes based upon PanglaoDB Augmented 2021 analysis of the 427 proteins with increased expression in liver homogenates on day-3 (D3) and day-6 (D6) post-RFA (from Enrichr). *Overlap* Number of identified potential associated proteins, *Adjusted p-value* Corrected for multiple testing, *Combined score* A composite metric integrating significance and enrichment magnitude. Overall, data confirms high likelihood of contribution of increased proteins from all 10 cell populations identified. *HSCs:*Hepatic stellate cells, *NK* Natural killer cells.

Table S4 Enrichment of pro-tumorigenic and proimmunogenic pathways across RNA, protein, and integrated RNA and protein datasets

	RNA only (N=443)			Protein only (N=427)			Enrichr - Combined RNA & Protein (N=797)				
**Name of Pathway**	**Classification**	**strength**	**signal**	**FDR**	**strength**	**signal**	**FDR**	**Index**	**p-value***	**OR**	**CS**
Positive regulation of leukocyte migration	GO:0002687	2.09	17.86	2.53E-102	2.09	11.57	4.13E-48	213	4.05E-09	18.3	405.3
Cytokine-mediated signaling	GO:0019221	1.77	11.9	2.00E-102	1.77	9.08	5.87E-63	11	4.71E-47	12.1	1363.9
Regulation of interleukin-10 production	GO:0032653	2.53	14.92	4.61E-41	2.53	10.6	5.73E-24	92	6.39E-15	19.0	689.4
Positive regulation of T cell proliferation	GO:0042102	2.29	16.7	7.21E-66	2.29	10.63	1.73E-30	47	7.42E-22	20.1	1064.5
Positive regulation of T cell activation	GO:0050870	1.95	13.3	1.69E-82	1.95	8.61	4.76E-37	63	1.75E-18	10.6	475.4
Chemokine-mediated signaling	GO:0070098	2.43	19.6	1.15E-71	2.43	6.83	2.32E-13	49	3.23E-21	28.2	1452.3
Chemokine receptors bind chemokines	MMU-380108	2.65	21.6	3.54E-66	2.65	8.56	8.26E-16	11	2.31E-34	43.2	3555.7
Signaling by Interleukins	MMU-449147	1.92	7.95	1.07E-33	1.92	10.4	2.86E-56	4	7.56E-63	10.1	1500.1
Cytokine Signaling in Immune system	MMU-1280215	1.74	8.33	9.24E-58	1.74	10.6	5.82E-90	6	1.78E-60	7.0	993.7
Cytokine-cytokine receptor interaction	mmu04060	1.89	19.66	6.79E-170	1.89	13.8	4.68E-100	1	2.05E-110	25.0	6458.9
JAK-STAT signaling	mmu04630	2.12	11.1	3.49E-42	2.12	13.8	9.25E-63	23	1.09E-23	9.5	522.7
Chemokine signaling	WP2292	2.1	12.4	3.34E-54	2.1	10.2	7.54E-38	6	6.43E-20	7.9	375.3
Regulation of endothelial cell proliferation	GO:0001936	2.09	15.41	2.54E-80	2.09	10.98	5.55E-44	59	8.02E-19	13.1	600.9

Transmembrane receptor protein tyrosine kinase signaling	GO:0007169	1.73	13.64	5.40 E-138	1.73	8.55	4.93E-63	9	7.77E-52	11.7	1451.4
Growth factor activity	GO:0008083	2.16	18.33	1.59 E-95	2.16	12.3	5.26E-48	3	3.53E-36	24.4	2115.7
Fibroblast growth factor receptor signaling	GO:0008543	2.56	13.49	3.83 E-34	2.56	7.59	2.93E-14	395	2.635 E-06	9.3	138.9
Positive regulation of MAPK cascade	GO:0043410	1.6	14.07	1.56 E-177	1.6	8	2.84E-77	6	1.61E-58	11.2	1559.6
Positive Regulation of Angiogenesis	GO:0045766	2.08	16.56	5.50 E-92	2.08	10.88	7.97E-44	81	8.97E-16	8.5	326.5
Positive regulation of epithelial cell proliferation	GO:0050679	1.96	15.07	6.28 E-100	1.96	10.91	1.85E-56	22	4.23E-29	15.1	1063.2
Positive regulation of PI3-Kinase/Prot Kinase B Signal Transduction	GO:0051897	2.21	15.39	1.49 E-65	2.21	9.69	3.03E-29	13	9.24E-46	17.5	1911.3
Regulation of ERK1 and ERK2 cascade	GO:0070372	1.8	15.81	3.64 E-147	1.8	9.98	2.33E-69	12	1.72E-46	13.4	1485.1
RAF/MAP kinase cascade	MMU-5673001	1.91	10.5	1.48 E-59	1.91	10.33	1.07E-57	41	2.23E-20	6.2	303.5
Signaling by Receptor Tyrosine Kinases	MMU-9006934	1.73	12.12	6.54 E-116	1.73	9.77	7.11E-81	7	2.73E-53	7.9	998.8
*Focal adhesion: PI3K-Akt-mTOR signaling***	WP2841	1.84	15.27	4.58 E-131	1.84	9.54	2.12E-58	1	9.53E-47	10.1	1116.5
Integrin-mediated signaling	GO:0007229	2.38	14.31	2.11 E-45	2.38	7.31	2.29E-15	54	4.30E-20	14.1	686.9
Extracellular matrix organization	GO:0030198	1.89	11.34	5.92 E-71	1.89	8.73	2.62E-43	38	2.62E-24	9.1	536.3

Positive regulation of fibroblast proliferation	GO:0048146	2.45	15.78	4.44 E-49	2.45	11.13	7.33E -28	166	2.60E -10	21.2	532.5
Integrin cell surface interactions	MMU-216083	2.48	27.15	8.34 E-113	2.48	9.11	1.58E -19	5	1.69E -60	59.0	8447.2
Extracellular matrix organization	MMU-1474244	1.95	17.26	5.76 E-128	1.95	10.39	3.18E -53	3	1.76E -64	14.1	2154.4
Integrin-mediated cell adhesion	WP6	2.34	10.64	1.84 E-28	2.34	8.44	2.72E -19	21	2.32E -09	6.6	143.1
Totals	average	2.09	14.81		2.09	9.89				16.04	1518.07
	STD	0.29	4.01		0.29	1.67				11.51	1821.23
	median			1.15 E-71			2.86E -56		2.31E -34		
	95% CI lower limit	1.6	7.95	1.56 E-177	1.6	6.83	4.68E -100		2.05E -110	15.76	138.94
	95% CI upper limit	2.65	27.15	1.84 E-28	2.65	13.8	2.32E -13		2.635 E-06	15.96	8447.15

Complementary data for the 30 moderate sized (49-545 genes) molecular pathways from the cytokine, growth factor, and collagen clusters (see Table 3) are presented. These confirm strong enrichment of these key pathways post radiofrequency ablation with demonstrated homology between the transcriptional and translational data. *N* Number of genes, *%* Percentage of total in group, *FDR* False discovery rate, *OR* Odds ratio, *CS* Combined score, *CI* Confidence interval, *STD* Standard deviation, *Strength* Enrichment strength score (from STRING database, STRING-db), *Signal* Enrichment signal strength. * Adjusted *p*-value from Enrichr, ** Enriched by STRING-db for both growth factor and collagen clusters, *** Significant for all 427 proteins.

Table S5 Identification of cell populations using gene markers.

Super Cluster	Clusters	Gene marker
Hepatocytes	11,5,2	Mup3, Apoa1, Hnf4a, Pck1
Kupfer cells	1,7	C1qa, Adgre1
NK cells	9	Klrb1c, Klrb1a
Neutrophils	18	Ccr1, Csf3r
Macrophages	8,12,14,17,19,22,25,26	Cd14, Fcgr3
B cells	6,13	Cd79a, Ms4a1
T cells	3	Thy1, Cd3d
HSCs	24, 10	Col1a1, Dcn, Loxl1, Mgp
Cholangiocytes	21,15	Sox9a, Krt19, Epcam, Krt7
Endothelial cells	0,4,23,16,20	Flt4, Bmp1, Adgrf5, Flt1

Table S6 Threshold robustness of proteomic analysis with sensitivity results and Wilson 95% CIs for proportions elevated at 15%, 25%, and 40%

Day	Threshold	Elevated (n)	Proportion Elevated	Median FC (elevated)	95% CI	Overlap with scRNA genes (n)
Day 3	15%	475	0.364	1.32	1.308–1.341	92
Day 6	15%	210	0.1609	1.28	1.258–1.326	27
Combined (either day)	15%	617	0.472796935	1.326	1.313–1.341	103
Day 3	25%	318	0.2437	1.41	1.388–1.429	63
Day 6	25%	123	0.0943	1.42	1.380–1.471	14
Combined (either day)	25%	413	0.316475096	1.417	1.398–1.432	73
Day 3	40%	166	0.1272	1.57	1.540–1.592	27
Day 6	40%	67	0.0513	1.57	1.511–1.695	7
Combined (either day)	40%	223	0.170881226	1.574	1.548–1.598	31

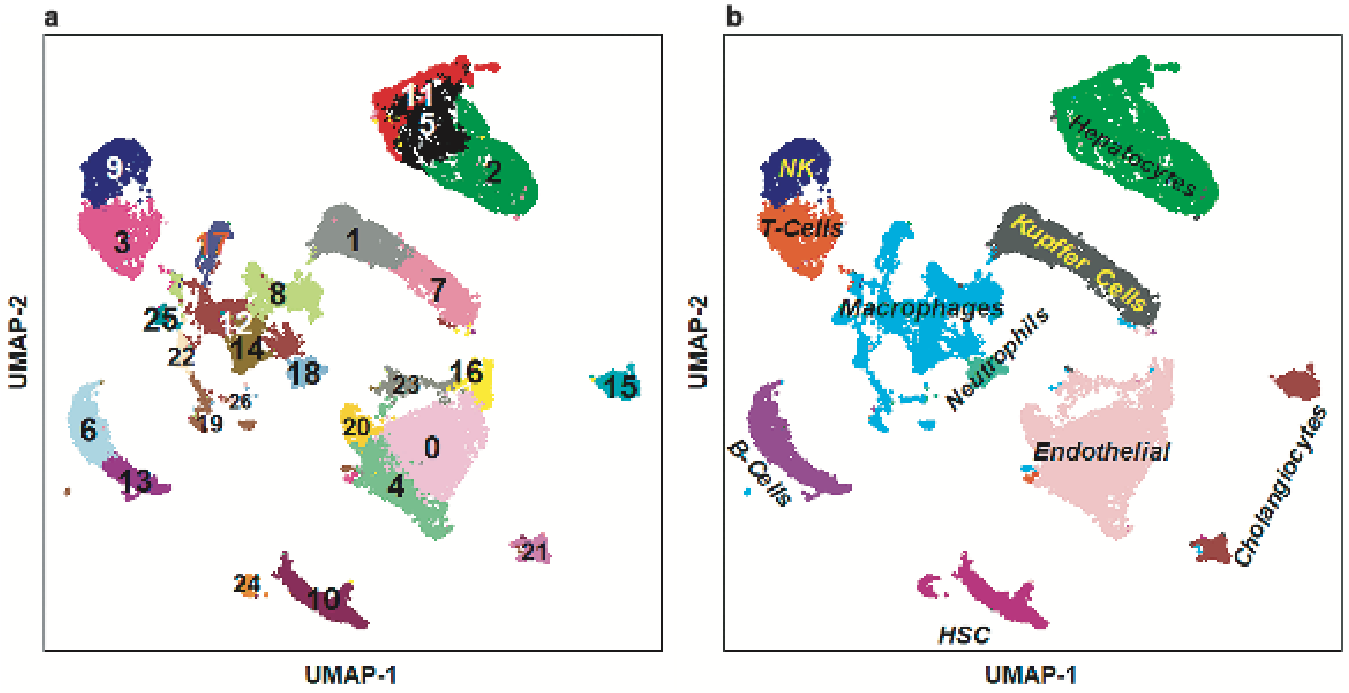

Fig. S1. Identification of cellular populations within the single-cell RNA sequencing samples as visualized by Uniform Manifold Approximation and Projection (UMAP). (**a**) 27 cellular clusters were identified across the three samples (0-26) arranged in descending order according to the number of cells. (**b**) These clusters were grouped into 10 cell populations based upon established gene markers (see Table S5). *HSC* Hepatic stellate cell, *NK* Natural killer cells.

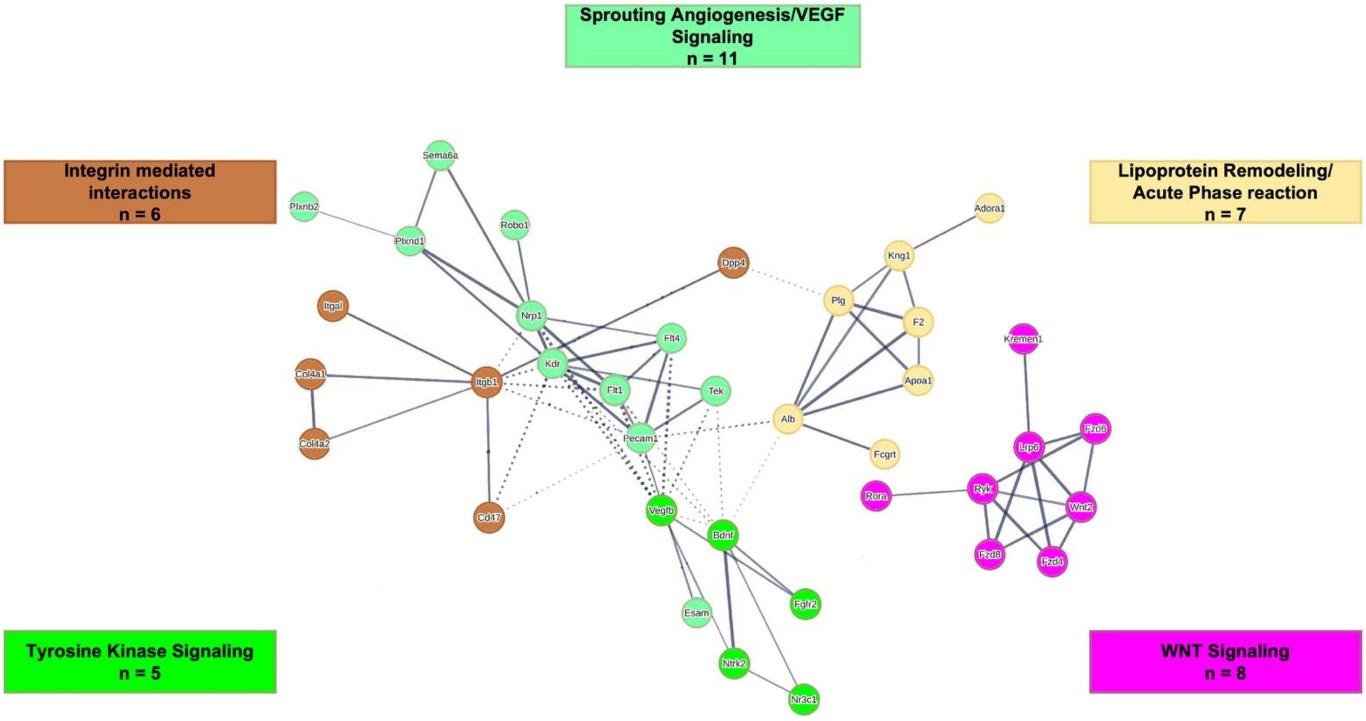

Fig. S2. STRING database pathway analysis of differentially expressed genes for new genes identified with increased expression on day-7 (D7) was performed on genes with increased expression (log2 fold change ≥ 1 in at least 1 cell population on day-7 (D7) exclusively (*n* = 83). Clustering yielded 5 clusters with a total of 37 genes in different functional protein groups, each containing ≥ 5 genes per cluster. The remaining 46 genes did not yield additional separate clusters of ≥ 5 genes or were not clustered at all and are therefore excluded from depiction.

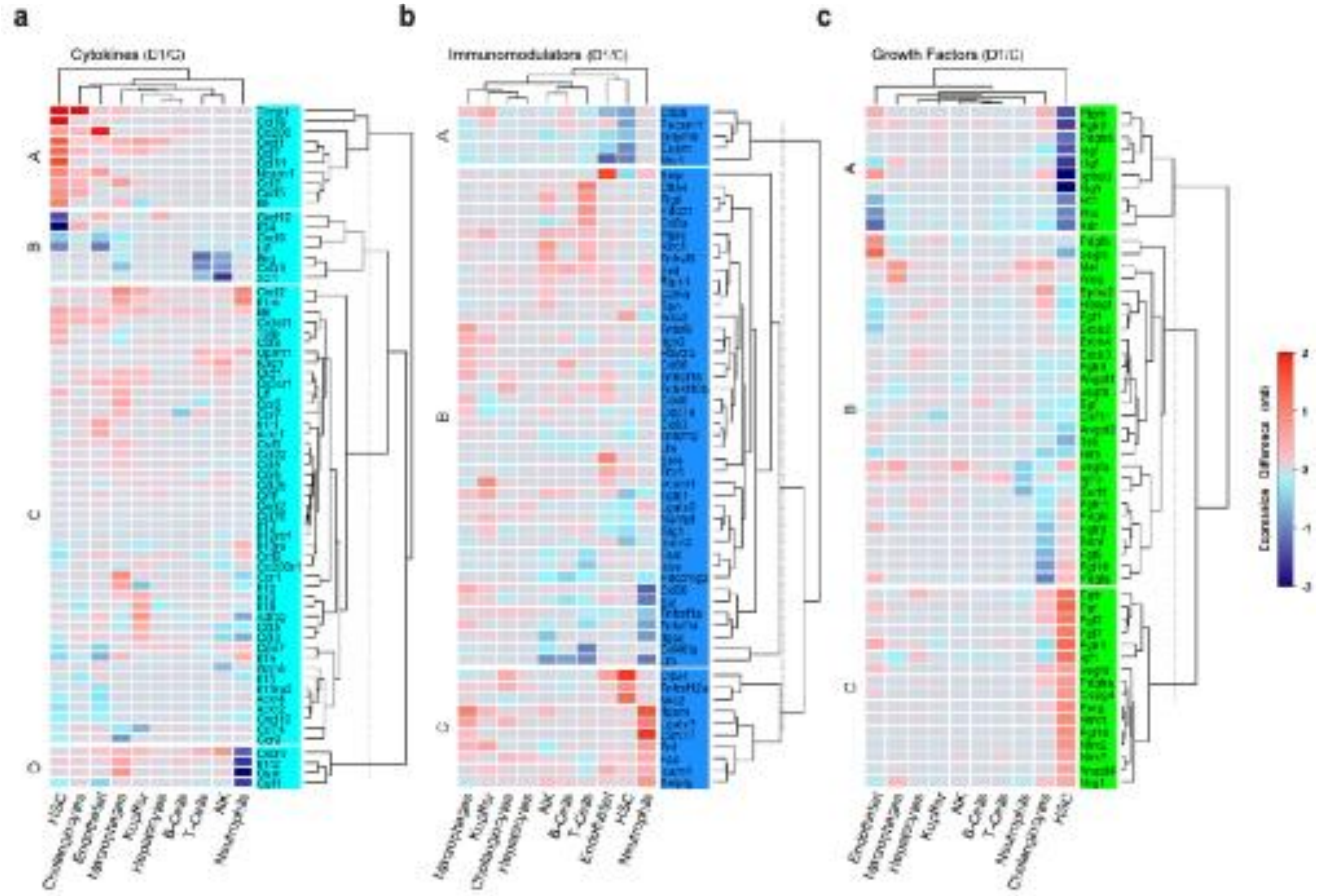

Fig. S3. Heatmaps comparing day-1 (D1) post-ablation and controls (C) for differentially expressed genes (DEGs) represented in the three largest protein clusters across all cell populations. Dendrograms display cell populations, arranged from left to right in descending order, based on the combined expression differences of all families, which were clustered using k-means. (**a**) Expression differences of DEGs in the cytokine group. (**b**) Expression differences of DEGs for the immunomodulators. (**c**) Expression differences of DEGs of the growth factor group. Noteworthy, DEGs in hepatic stellate cells (HSCs) in subfamily C showed the highest expression differences regarding growth factors. *NK* Natural killer cells, *STD* Standard deviation.

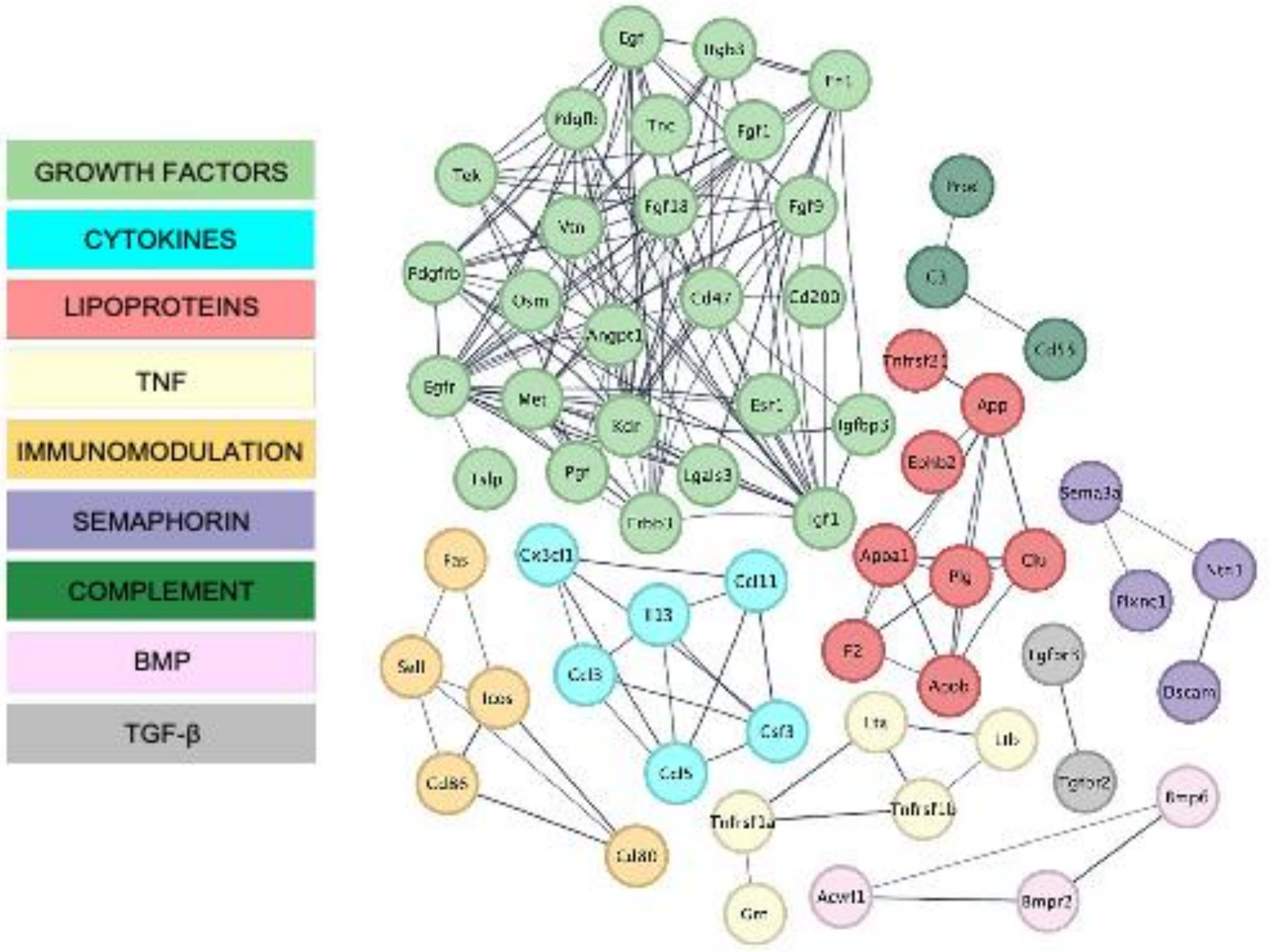

Fig. S4. Color-coded STRING database mapping of 73 genes elevated on both a transcriptional (single-cell RNA sequencing) and translational (proteomics) level. Markov clustering demonstrates a large number of growth factors (light green) highly associated with pro-tumorigenic angiogenesis, fibroblast, and endothelial proliferation pathways. Multiple proimmune associated clusters are simultaneously identified including cytokines (light blue), immunomodulators (orange), and tumor necrosis factor/interferon regulation (yellow).

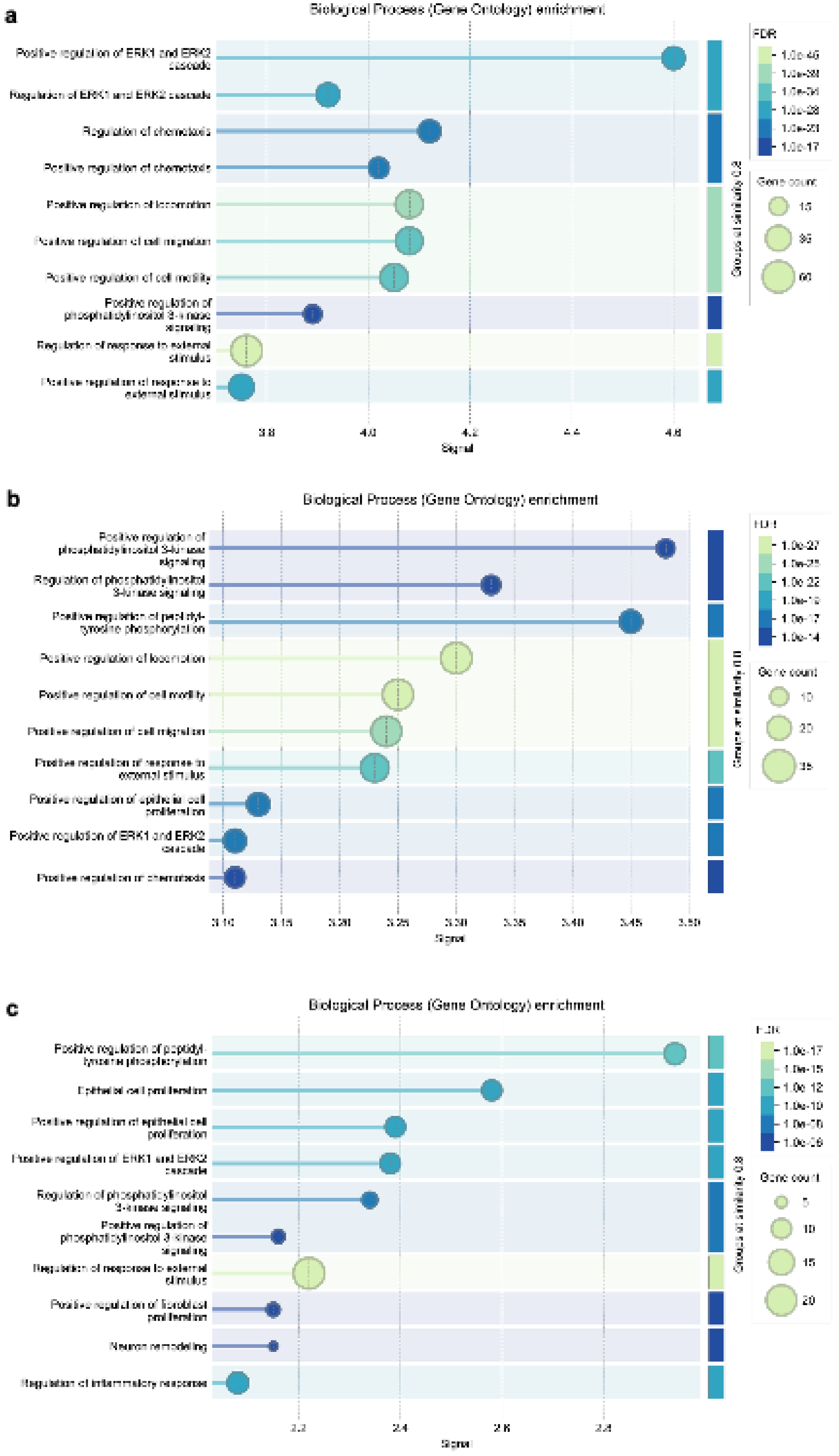
a
Biological Process (Gene Ontology) enrichment
Positive regulation of ERK1 and ERK2 cascade
Regulation of ERK1 and ERK2 cascade
Regulation of chemotaxis
Positive regulation of chemotaxis
Positive regulation of locomotion
Positive regulation of cell migration
Positive regulation of cell motility
Positive regulation of phosphatidylinositol 3-kinase signaling
Regulation of response to external stimulus
Positive regulation of response to external stimulus
3.8
4.0
4.2
4.4
4.6
Signal
Groups of similarity 0.8
FDR
Gene count
15
35
60
b
Biological Process (Gene Ontology) enrichment
Positive regulation of phosphatidylinositol 3-kinase signaling
Regulation of phosphatidylinositol 3-kinase signaling
Positive regulation of peptidyl-tyrosine phosphorylation
Positive regulation of locomotion
Positive regulation of cell motility
Positive regulation of cell migration
Positive regulation of response to external stimulus
Positive regulation of epithelial cell proliferation
Positive regulation of ERK1 and ERK2 cascade
Positive regulation of chemotaxis
Signal
Groups of similarity 0.8
FDR
1.0e-27
1.0e-22
1.0e-17
1.0e-14
Gene count
10
20
35
c
Biological Process (Gene Ontology) enrichment
Positive regulation of peptidyl-tyrosine phosphorylation
Epithelial cell proliferation
Positive regulation of epithelial cell proliferation
Positive regulation of ERK1 and ERK2 cascade
Regulation of phosphatidylinositol 3-kinase signaling
Positive regulation of phosphatidylinositol 3-kinase signaling
Regulation of response to external stimulus
Positive regulation of fibroblast proliferation
Neuron remodeling
Regulation of inflammatory response
2.2
2.4
2.6
2.8
Signal
Groups of similarity 0.8
FDR
1.0e-17
1.0e-15
1.0e-12
1.0e-10
Gene count
5
10
15
20

Fig. S5. STRING database mapping of the top ten gene ontology biological process (GOBP) pathways of overlapping proteomic and single-cell RNA sequencing results using 15% (**a**), 25% (**b**), and 40% (**c**) threshold of increased protein expression. The GOBP pathways for these thresholds show remarkable similarity to other pathways previously analyzed. using the threshold of log2 fold change ≥ 1 in at least 1 cell population.
